# Update on the role of R2R3-MYBs in the regulation of glucosinolates upon sulfur deficiency

**DOI:** 10.3389/fpls.2014.00626

**Published:** 2014-11-07

**Authors:** Henning Frerigmann, Tamara Gigolashvili

**Affiliations:** Department of Molecular Plant Physiology, BioCenter, Botanical Institute and Cluster of Excellence on Plant Sciences (CEPLAS), University of CologneCologne, Germany

**Keywords:** R2R3-MYB, *Arabidopsis thaliana*, glucosinolates (GSLs), regulation of glucosinolates, sulfur deficiency, SLIM1, MYB51, MYB28

## Abstract

To balance the flux of sulfur (S) into glucosinolates (GSL) and primary metabolites plants exploit various regulatory mechanisms particularly important upon S deficiency (−S). The role of MYB34, MYB51 and MYB122 controlling the production of indolic glucosinolates (IGs) and MYB28, MYB29, and MYB76 regulating the biosynthesis of aliphatic glucosinolates (AGs) in *Arabidopsis thaliana* has not been fully addressed at −S conditions yet. We show that the decline in the concentrations of GSL during S depletion does not coincide with the globally decreased transcription of *R2R3-MYBs*. Whereas the levels of GSL are diminished, the expression of *MYB34, MYB51, MYB122*, and *MYB28* is hardly changed in early phase of S limitation. Furthermore, the mRNA levels of these *MYBs* start to raise under prolonged S starvation. In parallel, we found that SLIM1 can downregulate the *MYBs in vitro* as demonstrated in *trans*-activation assays in cultured *Arabidopsis* cells with *SLIM1* as effector and *ProMYB51:uidA* as a reporter construct. However, *in vivo*, only the mRNA of *MYB29* and *MYB76* correlated with the levels of GSL at −S. We propose that the negative effect of SLIM1 on GSL regulatory genes can be overridden by a “low GSL signal” inducing the transcription of *MYBs* in a feedback regulatory loop. In accordance with this hypothesis, the expression of *MYB34, MYB51, MYB122*, and *CYP83B1* was further induced in *cyp79b2 cyp79b3* mutant exposed to −S conditions vs. *cyp79b2 cyp79b3* plants grown on control medium. In addition, the possible role of *MYBs* in the regulation of essential S assimilation enzymes, in the regulation of GSL biosynthesis upon accelerated termination of life cycles, in the mobilization of auxin and lateral root formation at S deficiency is discussed.

## Introduction

Sulfur (S) depletion leads to the decrease of the internal S levels, followed by a fast decrease in primary S-containing metabolites like glutathione as well as reduction in the levels of glucosinolates (GSLs) (Nikiforova et al., 2003, 2005; Hirai et al., 2004, 2005). Notably, the effects of S nutrition on GSL biosynthesis have been observed for years but the exact molecular mechanism by which changes in S supply modulate GSL metabolism are just starting to be understood. The backbone of GSLs contains from two to three S atoms, with one originating from 3′-phosphoadenosine 5′-phosphosulfate, the second one from glutathione, and the third being present in methionine derived aliphatic GSLs. This is the reason why the S status needs importantly to be regulated with GSL biosynthesis.

The analysis of transcript profile of *Arabidopsis thaliana* plants grown under S deficient conditions revealed the genes of the S assimilation pathway (sulfate transporters, cysteine biosynthesis, methionine biosynthesis and the glutathione cycle) upregulated in these plants after 48 h of S limitation (Hirai et al., 2003). Conversely, many genes of GSL biosynthesis were shown to be downregulated (Hirai et al., 2003; Maruyama-Nakashita et al., 2003; Nikiforova et al., 2003). Combining the metabolomic and transcriptomic studies demonstrates that S deficiency leads to reduced expression of all major GSL biosynthetic genes and, consequently, a reduction in GSL levels in plants. While decreasing the production of some S containing compounds, the plant maximizes uptake and utilization of S by increasing the expression of primary S assimilation genes.

In addition to changes in GSL biosynthesis rate, plants may also catabolize these secondary compounds. GSL catabolism has been postulated since the expression levels of genes coding for myrosinase-like proteins and thioglucosidases were upregulated in −S (Nikiforova et al., 2003, 2005; Hirai et al., 2004, 2005). During the myrosinase-catalyzed hydrolysis reaction (Bones and Rossiter, 1996; Rask et al., 2000), the GSL, which are normally stored in the vacuoles, need to come into contact (e.g., due to tissue disruption) with cytosolic myrosinases to be hydrolyzed. However, under conditions of −S, GSLs might be also degraded in intact plants by myrosinase-like proteins (Schnug and Haneklaus, 1993; Schnug et al., 1995), which do not require tissue damage. Similar mechanism of GSL degradation has been reported to be important for the plant innate immunity (Bednarek et al., 2009). S released after *in vivo* GSL hydrolysis can be further incorporated into essential S-containing compounds and therefore maintain the vital processes in plant metabolism. Activation of GSL catabolism at −S is among the processes of −S response, which are least understood and cannot be explained directly by flux alterations because of changed concentrations of S-containing compounds (Hoefgen and Nikiforova, 2008). Along with the release of S, the hydrolysis of indolic GSLs (IGs) allows an increased synthesis of auxin which promotes lateral root formation and facilitates in this way the uptake of sulfate. Although the accumulation of auxin has not been shown to be induced in S-depletion experiments, several observations suggest the hydrolysis of IG upon this condition. These include an activation of genes involved in synthesis of tryptophan (Nikiforova et al., 2003), an activated GSL catabolism (Nikiforova et al., 2003, 2005; Hirai et al., 2004, 2005) and strong overexpression of nitrilases (Kutz et al., 2002). Additionally to that Nikiforova et al. (2003) reported transcriptional activation of genes involved in synthesis of indolic glucosinolates (IGs), downstream genes leading to auxin and its derivatives pointing to a possible flux to IAA biosynthesis under −S conditions.

**Figure 1:**
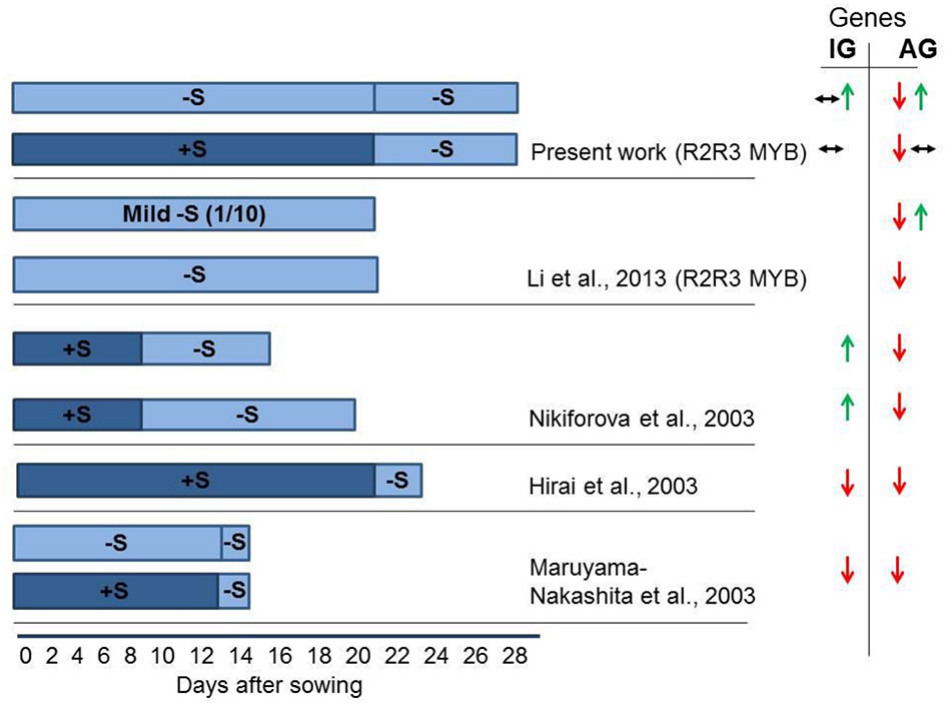
**Experimental scheme of sulfur (S) starvation showing number of days at S deficient conditions for four independent experiments conducted in different groups (Hirai et al., 2003; Maruyama-Nakashita et al., 2003; Nikiforova et al., 2003; Li et al., 2013; and present work)**. The seeds were either sown directly in −S medium (bright blue), or were grown in S sufficient (dark blue) medium followed by transfer to −S medium. This figure indicates that the duration of S deficiency determines the possible outcome on GSL gene expression. The S deficiency of 24–48 h (Hirai et al., 2003; Maruyama-Nakashita et al., 2003) was shown to inhibit GSL gene expression. The S deficiency applied for 7 days (present work) revealed absence of change in the expression of MYBs regulating IGs or downregulation of MYBs regulating AGs. The mild S shortage (1/10 of S levels) for 21 days revealed the activation of *MYB28* along with the downregulation of *MYB29* and *MYB76* (Li et al., 2013). The long-term depletion of S (Nikiforova et al., 2003—13 days; Present work—growth at −S for 28 days) revealed the activation of *MYB34, MYB51, MYB122* and *MYB28* and downregulation of *MYB29* and *MYB76*. Green arrows show increased gene expression. Red arrows show decreased gene expression. Black arrows indicate no significant change in the expression of gene. Two types of arrows shown simultaneously are indicative for the downregulation (↓), upregulation (↑), or no changes (↔) in the expression of different genes in GSL biosynthesis or regulation. As we do not possess original expression profiling data (Hirai et al., 2003; Maruyama-Nakashita et al., 2003; Nikiforova et al., 2003), the changes shown in this figure can be applied only to some selected genes discussed by the authors of original manuscripts. Hirai et al. (2003), Maruyama-Nakashita et al. (2003), Nikiforova et al. (2003) discuss only the expression of some genes in GSL biosynthesis, whereas Li et al. (2013) discuss the expression of R2R3-MYB regulators.

Figure 1 summarizes finding of Hirai et al. (2003), Maruyama-Nakashita et al. (2003), Nikiforova et al. (2003), Li et al. (2013) on the effect of S deficiency of the transcription of GSL genes. The duration of S deficiency appears to determine the outcome of the gene expression, as S deficiency of 24–48 h duration was shown to inhibit gene expression, whereas under long-term depletion of S (lasting for 7 days or more), the activation of some GSL biosynthetic genes is registered. Remarkably, Li et al. (2013) has recently reported the activation of *MYB28* as measured after 3 weeks of mild −S conditions.

**Figure 2:**
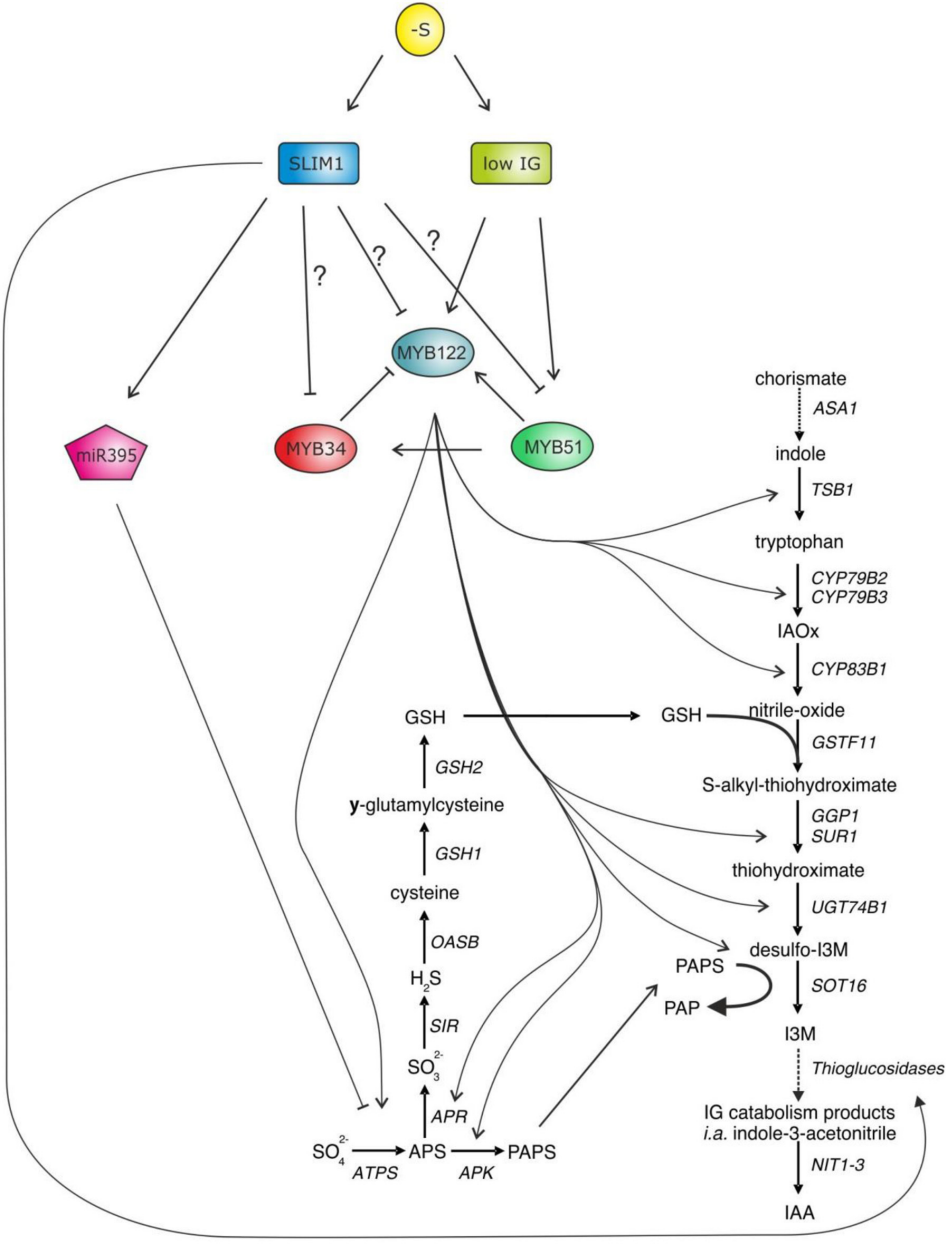
**MYB34, MYB51, and MYB122 are the central regulators of IG biosynthesis in *Arabidopsis* controlling transcription of the genes of core GSL biosynthesis and primary S assimilation**. As accumulation of GSL is strongly diminished upon −S, it has been thought that these three *MYBs* along with the MYBs regulating aliphatic GSLs (*MYB28, MYB29*, and *MYB76*—not shown) need to be negatively regulated upon S deficiency. The SLIM1—a well-known regulator in S deficiency response has been suggested as an upstream negative regulator of *R2R3-MYBs* (Takahashi et al., 2011). In addition, SLIM1 seems to be able to stimulate GSL degradation by activating myrosinase-like proteins of thioglucosidases, which are able to degrade GSL and release the S. However, during this enzymatic hydrolysis of GSL, plants additionally release auxin, which is an important hormone to accelerate the lateral root formation, which can allow plant to acquire more sulfate.

The primary and secondary S assimilation is positively controlled by the group of R2R3-MYB transcription factors, which are also known to regulate GSL biosynthesis (Hirai et al., 2007; Sønderby et al., 2007; Gigolashvili et al., 2007a; Yatusevich et al., 2009) (Figure 2). There are 6 different MYBs involved in GSL regulation, with MYB34, MYB51, and MYB122 controlling the production of IGs and MYB28, MYB29, and MYB76 controlling the production of aliphatic glucosinolates (AGs). Remarkably, these MYBs can also stimulate expression of primary S assimilation enzymes, enhancing substrate supply for GSL biosynthesis. Although all six MYB factors regulate adenosine-5′-phosphosulfate reductase (APR) and adenosine-5′-phosphosulfate kinase (APK), the trans-activation of ATP sulfurylase (ATPS) was isoform specific in relation to the aliphatic and indolic group.

S-deficiency response is largely controlled by Sulfur Limitation 1 (SLIM1). SLIM1 is a key transcriptional regulator of sulfate uptake identified from a genetic screen for *Arabidopsis* mutants disrupted in the S-limitation response. SLIM1 is the first transcription factor suggested to regulate plant metabolism upon −S, by, e.g., activating the sulfate acquisition (Maruyama-Nakashita et al., 2006). In addition, SLIM1 can probably activate the above described GSL catabolism process (like a putative thioglucosidase) and has been suggested as a negative regulator of *R2R3-MYB* genes controlling production of GSL in plants (Figure 2). In the support of this hypothesis, it was shown that the −S dependent decline in the expression of *MYB34* was not present any more in *slim1* knockout, pointing to the role of SLIM1 as a negative regulator of *MYB34* upon S limitation. The effect of SLIM1 on regulation of other *MYBs* was unclear (Maruyama-Nakashita et al., 2006). Still, a recent review on S assimilation in plants has suggested that *R2R3-MYBs* can be regulated by SLIM1 to repress the GSL biosynthesis (Takahashi et al., 2011). Despite the link that seems to exist between S and the biosynthesis of GSL (Mugford et al., 2009, 2010, 2011; Yatusevich et al., 2010; Kopriva et al., 2012; Huseby et al., 2013), the molecular mechanisms remain less clear. Particularly little is known about the role of R2R3-MYBs in the S-deficiency mediated regulation of GSL biosynthesis in *Arabidopsis*.

We show that SLIM1 has a potential to downregulate the expression of *R2R3-MYBs* regulating GSL biosynthesis *in vitro*. However upon sulfate deficiency, the mRNA levels of main aliphatic and indolic GSL regulators *MYB28, MYB34, MYB51*, and *MYB122* are either not changed or increased. To explain this observation we suggested that the negative effect of SLIM1 on GSL regulatory genes can be overridden by a “low GSL signal” inducing the transcription of *MYBs* in a feedback regulatory loop.

## Results

### SLIM1 is capable of repressing the transcription of *R2R3-MYBs* in cultured *Arabidopsis* cells *in vitro*

**Figure 3:**
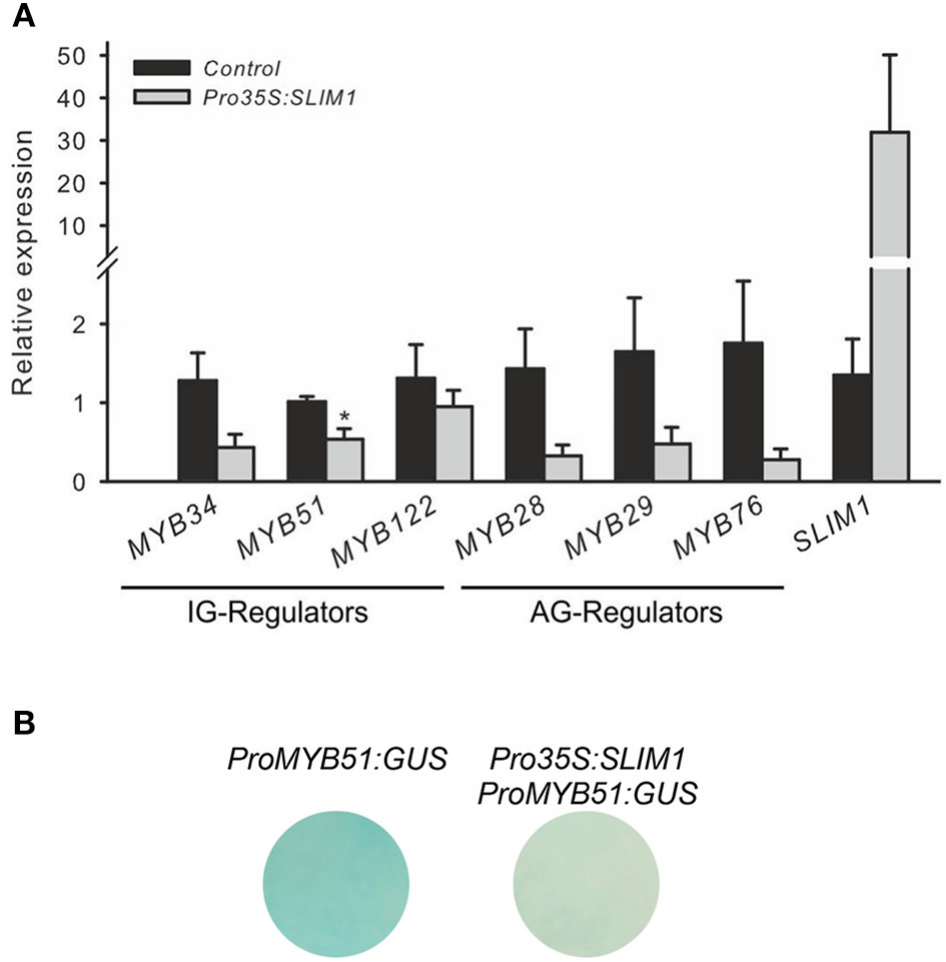
**SLIM1 is able to suppress the expression of *R2R3-MYBs*. (A)** Expression of *R2R3-MYBs* regulating the biosynthesis of IG (*MYB34*, *MYB51*, *MYB122*) and AG (*MYB28*, *MYB29*, *MYB76*) in cultured *Arabidopsis* cells overproducing *SLIM1*; Data for *MYB28, MYB29* and *MYB76* are presented as means ± *SE* from five independent biological replicates (*n* = 5). Data for *MYB51, MYB34*, and *MYB122* are presented as means ± *SE* from 7 independent biological replicates (*n* = 7). Values marked with asterisks are significantly different from controls (+S to +S) (Student's *t*-test; *p* < 0.05). **(B)** Expression of promoter *ProMYB51:GUS* is suppressed by *Pro35S:SLIM1* in cultured *Arabidopsis* cells. This experiment was replicated twice with three independent biological replicates. Promoter of *MYB51* is the only MYB showing strong activity in cultured cells. Other *MYBs* regulators controlling the production of IG and AG show no staining in cultured cells, which hampered the potential visualization of inhibitory effects of SLIM1 on *MYBs*.

The S-deficiency regulator SLIM1 is an important regulator of −S response, which activates the sulfate acquisition and probably GSL catabolism with latter releasing S from these S-rich compounds. In addition, SLIM1 has been suggested to repress the GSL biosynthesis, probably by repressing the *R2R3-MYBs* which control their biosynthesis. To study how SLIM1 affects expression of *MYBs*, qRT-PCR analysis of *MYBs* in cultured *Arabidopsis* cells transiently over-expressing *SLIM1* was conducted. Figure 3A shows that *SLIM1* is capable of repressing the expression of *MYB34, MYB51, MYB28, MYB29*, and *MYB76 in vitro*.

Further SLIM-MYB interactions were performed with the help of *trans*-activation assay (Berger et al., 2007). In brief, the co-transformation assay with *Pro35S:SLIM1* as an effector construct and promoters of *R2R3-MYBs* as reporter was conducted. To be able to observe the repressing activity of SLIM1 on *MYBs in trans*, the promoter used in the assay should be strongly expressed in cultured cells. Among all promoters tested only *ProMYB51:GUS* showed strong GUS staining allowing the usage of *ProMYB51* in this assay. As shown on Figure 3B, the interaction of *Pro35S:SLIM1* with the *ProMYB51:GUS* revealed an inhibitory effect of *SLIM1* on the expression of *ProMYB51:GUS* cultured *Arabidopsis* cells (Figure 3B), confirming the potential of SLIM1 to repress transcription of *MYBs*. Despite the insights on the inhibitory role of SLIM *in vitro*, it's role on the expression of *R2R3-MYBs in vivo* remains to be studied in future.

### *R2R3-MYBs* regulating glucosinolate biosynthesis are differently responding to −S

**Figure 4:**
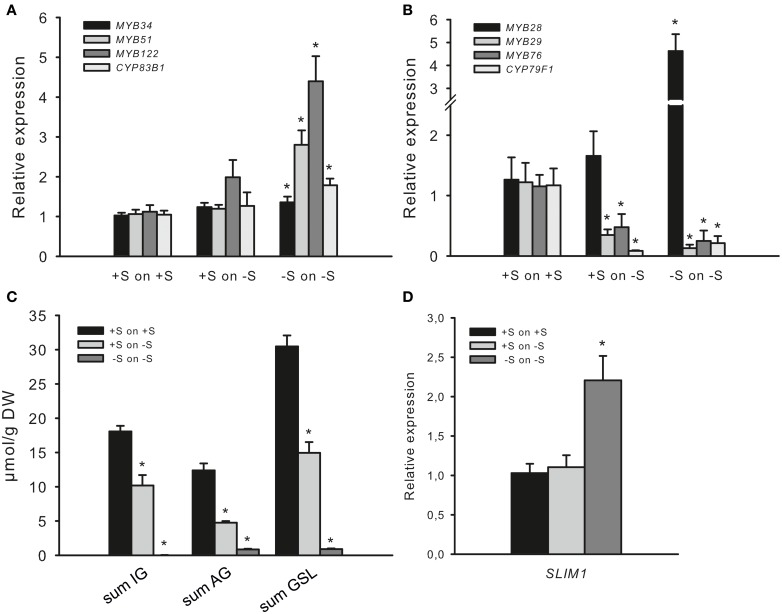
**Sulfur deficiency differently affects the expression of *R2R3-MYBs*. (A)** Relative transcript levels of *MYB34*, *MYB51*, *MYB122*, and *CYP83B1*; **(B)** Relative transcript levels of *MYB28*, *MYB29*, *MYB76*, and *CYP79F1*; **(C)** Accumulation of indolic (IG) and aliphatic (AG) glucosinolates; **(D)** Relative transcript levels of *SLIM1*. Transcript levels of *R2R3-MYBs*, GSL biosynthesis genes (*CYP83B1* and *CYP79F1*) and *SLIM1* and GSL contents were determined by qPCR or UPLC analysis. Three-week-old seedlings grown on +S or −S plates were transferred to plates with +S and −S, respectively, grown for 7 more days followed by the analysis of gene transcript levels **(A,B,D)** and the sum of IG, AG, and GSL levels **(C)**. Relative gene expression values are given compared to plants transferred from +S to +S (+S to +S = 1). Data are presented as means ± *SE* from four independent cultivations with three biological replicates (*n* = 12). **(C)** For GSL analysis, two independent cultivations with four biological replicates were done (*n* = 8). GSLs were totalled either as the sum of IG (I3M, 4MO-I3M, 1MO-I3M), sum of AG (3MSOP, 4MSOB, 5MSOP, 8MSOO) or sum of all GSL (IG plus AG). Values marked with asterisks are significantly different from controls (+S to +S) (Student's *t*-test; *p* < 0.05).

To study the adaptive changes in GSL accumulation and MYB regulation upon S deficiency, wild-type seedlings of *Arabidopsis* were seeded out on Hoagland's media (+S) on agar plates and for the analyses of S deficiency were further cultivated on −S plates. Three-week-old *Arabidopsis* seedlings grown on +S or −S plates were transferred either to plates with the −S, or to new plates that maintained the existing S growth conditions and used for the analysis of expression levels of *MYBs* and GSL accumulation after 7 days of exposure to −S. This approach enabled us to describe the changes in the *MYB* expression after 7 and 28 days of S depletion conditions. Plants grown at −S for 7 days (“+S to −S”) do not display any obvious symptoms, whereas plants grown for 28 days (“−S to −S”) were retarded in growth. Plants transferred from +S to +S served as a control for the possible induction of genes by mechanical stress and were used as a calibrator for the relative expression analysis of *MYBs*. Figure 4 shows that the expression levels of *MYBs* regulating IGs is not changed in seedlings transferred from +S to −S condition, (Figure 4A), although the amounts of IGs are significantly reduced (Figure 4C). Moreover, when Arabidopsis seedlings were transferred from −S to −S, these plants showed significantly increased expression of *MYB34*, *MYB51* and *MYB122* along with the further decreased levels of IGs. Notably, changes in the expression of *MYBs* went along with the increased expression of *CYP83B1* in “−S to −S” plants (Figure 4A). Thus, in “+S to −S” and “−S to −S” plants we have uncoupling of *MYB* and *SLIM1* (Figure 4D) transcripts with and GSL (Figure 4C) accumulation levels pointing on additional regulatory signal interfering with negative regulation of GSL regulation.

Not only the mRNA levels of *MYBs* regulating IG biosynthesis but also the mRNA of *MYBs* regulating AGs were different at “+S to −S” and at “−S to −S” conditions. The accumulation of AGs was significantly reduced in seedlings transferred from +S to −S and even further reduced in “−S to −S” plants (Figure 4C), which went along with the significantly declined mRNA levels of *MYB29, MYB76* and AG biosynthesis gene *CYP79F1* in both conditions (Figure 4B). Furthermore, the transfer of seedlings from +S to −S did not negatively affect the expression of *MYB28*, whereas “−S to −S” plans manifested significantly increased level of *MYB28*. This observation also indicates uncoupling of *MYB28* transcript with the level of GSL, pointing to additional regulatory signal taking over the “−S signal” aiming the negative regulation of GSL biosynthesis.

Altogether our observation on the expression of *MYBs* suggest an existence of more complex SLIM1-independent signaling, which can positively regulate transcription of *MYB34*, *MYB51*, *MYB122*, and *MYB28* along with the negative regulation of *MYB29* and *MYB76* at −S. It's probably not a coincidence that the expression of two latter regulators of AG biosynthesis was significantly decreased at −S. One might suggest that AGs containing one more molecule of S need to be more tightly regulated at −S. This mechanism will probably allow to channel S into essential for the plant survival metabolites. In accordance with this suggestion the levels of AGs were strongly diminished after 7 days of −S than the levels of IGs. Still, this logic does not explain an increased expression of *MYB28* under the same condition and point to a specific role of *MYB28* at −S, differing from the role of MYB29 and MYB76.

Along with the interesting insights on the different regulation of MYBs regulating IG and AG biosynthesis we suggested that an additional regulatory signal positively controlling the transcription of *MYB34, MYB51*, and *MYB122* might be a “low IG level” in plants.

### Increased expression of *MYB34*, *MYB51*, and *MYB122* upon −S can result from the low levels of IGs triggering the transcription of these *MYBs* in negative feedback loop

**Figure 5:**
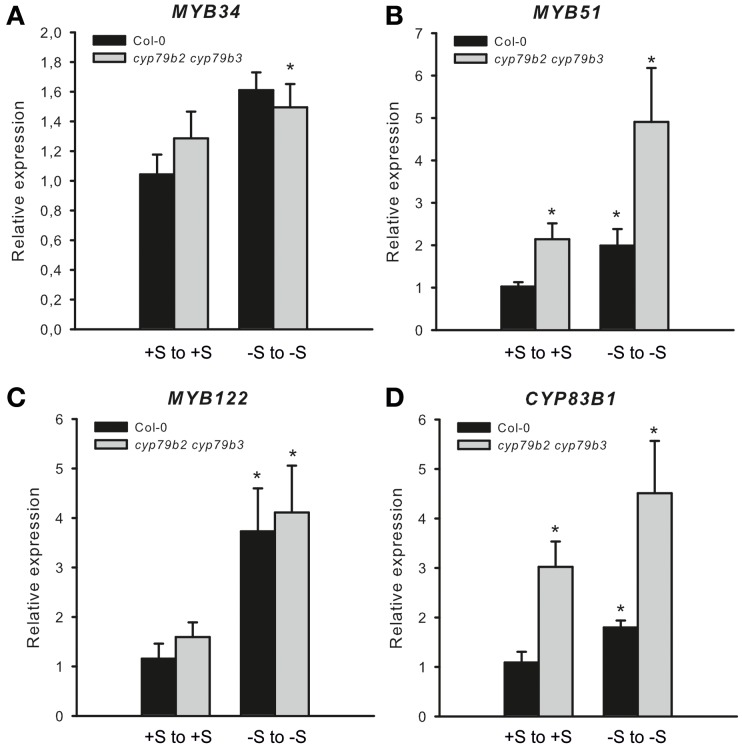
**Expression of *MYB34*, *MYB51*, and *MYB122* in *cyp79b3 cyp79b3* mutant deficient in IG biosynthesis**. The relative expression of *MYB34* **(A)**, *MYB51* **(B)**, *MYB122* **(C)**, and *CYP83B1* **(D)** was measured in wild-type plants and *cyp79b2 cyp79b3* knockout mutant (Col-0 from +S on +S = 1). Data are presented as means ± *SE* from two independent cultivations with three biological replicates (*n* = 6). Values marked with asterisks are significantly different from the control (Col-0 from +S on +S) (Student's *t*-test; *p* < 0.05).

To address whether low IGs may have signaling function we analyzed the transcript levels of *MYB34, MYB51* and *MYB122* and of IG biosynthetic gene *CYP83B1* in the *cyp79b2 cyp79b3* mutant (Zhao et al., 2002). This mutant is known to be devoid of IG and therefore an increased level of *MYBs* especially at “−S to −S” was expected. Figure 5 shows elevated expression of *CYP83B1* in *cyp79b2 cyp79b3* mutant already on +S to +S medium, together with an induction of *MYB51*. This observation supports previous findings on the negative feedback regulation of IG biosynthesis driven by low IG levels in IG deficient mutants like *cyp83b1, atr1* and *cyp83b1 atr1* (Celenza et al., 2005). Furthermore, the *cyp79b2 cyp79b3* mutant plants revealed a further increase in *MYB51*, *MYB122* and also of *CYP83B1* expression levels at “−S to −S” conditions. The mRNA levels of *MYB34* were also increased at “−S to −S” but only moderately. In sum, analysis of *MYB34, MYB51* and *MYB122* expression in *cyp79b2 cyp79b3* mutant plants grown at “−S to −S” indicated the possible role of “low IGs signal” in triggering the transcription of these transcription factors (TFs).

## Discussion

The biosynthesis of S-containing GSLs competes with primary S metabolism. SLIM1 is a S-deficiency induced TF, which was correlated with the induction of transcriptional changes leading to a downregulation of GSL biosynthetic genes and with the induction of genes involved in GSL catabolism (Maruyama-Nakashita et al., 2006). A decrease in the steady-state levels of *MYB34* in microarray experiments of wild-type vs. *slim1* mutants exposed to S deficiency (Maruyama-Nakashita et al., 2006) suggests that *MYBs* could be negatively regulated by SLIM1. Based on this observation and on the fact that GSL biosynthesis is negatively regulated at −S, a recent review work has summarized that MYB transcription factors should be negatively regulated by S limitation (Takahashi et al., 2011).

Still, the regulation of R2R3-MYBs at −S has not been specifically addressed. Furthermore, the recent finding of Li et al. (2013) reported counterintuitive results showing significantly increased expression of *MYB28* upon mild S deficiency. To find out how *R2R3-MYBs* controlling IG and AG biosynthesis are regulated upon S deficiency conditions, the expression of the *MYB34, MYB51*, and *MYB122* on one side and of the *MYB28, MYB29*, and *MYB76* on other side was analyzed at two different −S conditions.

### Regulation of *R2R3 MYBs* expression at −S

**Figure 6:**
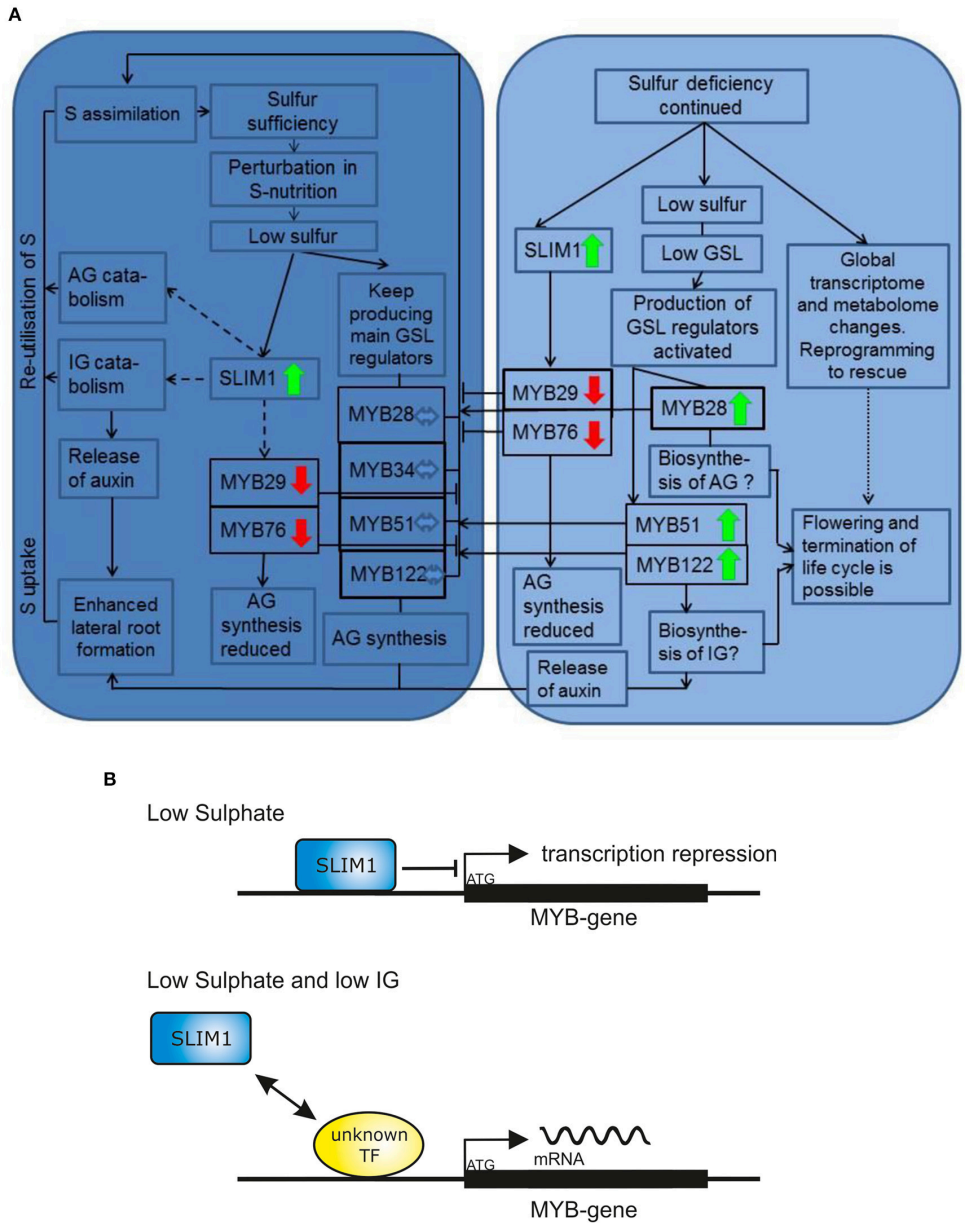
**The final model for the R2R3-MYB-mediated regulation of glucosinolate biosynthesis upon sulfur stress. (A)** The early (7 days of −S; the dark blue chart) and late (28 days of −S; the bright blue chart) sulfur stress responses including changes in plant metabolism and expression of *R2R3-MYBs*. Only mRNA levels of *MYB29* and *MYB76* are downregulated in both early and late S deficiency responses. The expression of *MYB28, MYB34, MYB51*, and *MYB122* is not changed after 7 days of −S. The expression of *MYB28, MYB51*, and *MYB122* is significantly increased after 28 days of −S deficiency indicating that the inhibitory signal of SLIM on *MYBs* is overridden by “low GSL signal” (shown in detail in Figure 6B). Solid lines/arrows indicate positive (inducing) effects; Solid or dashed lines with an aslant dash indicate negative (inhibiting) effects; Dashed lines/arrows indicate postulated pathways; Dotted lines/arrows indicate complex changes with many and not highlight pathways affected. Bold green arrows indicate increased expression of *R2R3-MYB* gene. Bold red arrows indicate decreased expression of *R2R3-MYB* gene. **(B)** Model explaining the increased expression of *MYBs* under continuous sulfur deficiency. The SLIM inhibitory effect can be overridden by “low GSL signal,” which positively regulates GSL biosynthesis with a so far unknown TF or regulatory switch by a negative feedback mechanism. This work demonstrated, that at −S low IGs can activate the expression of MYBs regulating their production.

Whereas both 7 days of “+S to −S” and 28 days of “−S to −S” caused drastic decrease in GSL accumulation, the *R2R3 MYBs* responded differently to these −S conditions. The *MYB29* and *MYB76* were repressed in both −S conditions and correlated with the levels of GSL indicating the positive “feed-forward regulation” of these two genes at −S. Conversely, the expression of *MYB28, MYB51, MYB34*, and *MYB122* was not affected after 7 days of −S but was significantly increased after 28 days. The increased levels of mRNA of *MYBs* were counterintuitive, as we expected to find the correlation of the levels of GSL with the expression levels of *MYBs*. To explain this finding we suggested that a specific signal (activated by −S but SLIM1 independent) is interfering in negative GSL regulation and activates expression of *MYBs* (Figure 6A) for the reasons discussed below.

Several possible scenarios explaining high expression of *MYBs* under −S conditions could be suggested. Firstly, we propose that the downregulation of IG biosynthesis at −S could be overridden by “a low IG signal” inducing the transcription of *MYB51* and *MYB122* in a feedback regulatory loop. In analogy, “a low AG signal” can induce the transcription of *MYB28* in a feedback regulatory loop. The transcription of *MYB28, MYB51*, and *MYB122* is, therefore, increased to push the production of these compounds when GSL go below a certain threshold limit (summarized in Figure 6A). Remarkably, and in accordance with this hypothesis, the expression of *MYB34, MYB51*, and *MYB122* and of IG biosynthesis gene *CYP83B1* was further induced in *cyp79b2 cyp79b3* mutant exposed to −S conditions vs. *cyp79b2 cyp79b3* plants grown on Hoagland's medium (Figure 5). Secondly, the activation of these *MYBs* at −S could be important for plants because they regulate essential S assimilation enzymes like APR and APK. The role of MYBs in the specific regulation of these enzymes (e.g., APR) could be of special importance upon −S. Especially because the *APR* genes are known to be independent from SLIM1 and can be therefore controlled by MYBs. To further address this hypothesis, the primary S assimilation of plants need to be studied at −S in plants devoid of major *MYBs* (e.g., *myb28 myb51* and/or *myb28 myb29 myb34 myb51*). Thirdly, it is also possible that at continuous −S when the metabolism of plants exposed to stress is reprogrammed to the shortening of the life cycle and speeding up the seed production (Hoefgen and Nikiforova, 2008). Among the numerous changes happening in the plant metabolism are positive changes in the expression of *MYBs* required to produce GSL for the seeds (Figure 6A). In this case the MYB28 and MYB51 could be the important TFs taking over the responsibility to control the synthesis and the transport of GSL into the seeds ensuring the survival of plant offspring. Notably, this hypothesis is not in line with the previous observations showing decline in the expression of *MYB28* (Gigolashvili et al., 2007b) and *MYB51* (Gigolashvili et al., 2007a) on the onset of bolting. Conversely, the expression of genes closely related to MYB28 is strongly increased with the onset of flowering in *Brassica juncea* (Augustine et al., 2013). To verify this hypothesis it will be necessary to analyse GSL accumulation in seeds of triple *myb28 myb51 myb122* mutant “forced” to complete their life cycle at −S. Finally and according to the fourth scenario, which can explain the activation of *MYBs* controlling IG production, the positive regulation of *MYB34*, *MYB51*, and *MYB122* could be an important mechanism allowing the plant to produce auxin either via IAOx or via catabolism of IG (Figure 6A) with the involvement of nitrilases (Kutz et al., 2002). In conformity with this scenario, the levels of IG are stronger declined than the levels of IG in “−S to −S” plants (Figure 4C). To prove this hypothesis the catabolism of IG *in vivo* at −S needs to be addressed in more detail. Alternatively, accumulation of auxin at −S in triple *myb34 myb51 myb122* mutant in comparison to wild-type plants needs to be analyzed.

One more possible scenario, which can explain the up-regulation of *MYB28* along with the down regulation of *MYB29* and *MYB76* at −S has been recently discussed by Li et al. (2013). This hypothesis is based on the observation that MYB mutually regulate each other and therefore an increased transcription of *MYB28* may result from the decreased transcription of *MYB29* and *MYB76* (Sønderby, 2010). However, even if this hypothesis is applicable to AG pathway, it cannot be applied to explain the upregulation of *MYB34, MYB51*, and *MYB122*, at “−S to −S” conditions.

Even if each of the considered hypothesis can explain the observed positive regulation of *R2R3-MYBs* at −S alone, the GSL-S balance in plants is probably controlled in a complex combinatorial network integrating many signals (Figure 6A). It can be therefore assumed that several of suggested scenarios can be happening simultaneously.

### Feedback regulatory loop in GSL biosynthesis as a trigger activating transcription of *R2R3-MYBs* under S deficiency

Our first hypothesis suggested that negative feedback regulation of GSL is switched upon low GSL status in the cell, resulting in the activation *MYB28, MYB51*, and *MYB122* at −S. Low-GSL signal activating the transcription of *MYBs* seem to act together but against the SLIM1 to regulate the glucosinolate-sulfur balance in the cell (Figure 6B). In agreement with this hypothesis, the expression of *MYB34, MYB51*, and *MYB122* and of IG biosynthesis gene *CYP83B1* is induced in *cyp79b2 cyp79b3* mutant vs. wild-type plants and is further stimulated in *cyp79b2 cyp79b3* plants exposed to −S conditions (Figure 5).

It was also previously reported that *Arabidopsis* plants possess a mechanism reacting to low levels of GSLs as a signal for induction of their synthesis (Smolen and Bender, 2002; Mugford et al., 2009). For example, the negative feedback regulation of the IGs was shown in *cyp83b1* mutant having increased levels of *ASA1, TSB1, CYP79B2, CYP79B3*, and *MYB34* transcripts, suggesting feedback inhibition of expression of *MYB* regulators by IGs (or their intermediates) (Smolen and Bender, 2002; Celenza et al., 2005). Furthermore, expression of *CYP83B1* and *MYB34* was significantly enhanced in IG deficient *cyp79b2 cyp79b3* double mutant, substantiating the existence of a feedback regulatory loop (Celenza et al., 2005). Finally, the *CYP79B2* and *CYP79B3* transcripts were shown to be suppressed in the *myb34 cyp83b1* (*atr1-2 cyp83b1)* double mutant indicating the MYBs to be an important element of the negative feedback loop. It has been recently suggested that GSL may bind MYB transcription factors and thereby modulate their activity toward to promoters of biosynthetic genes (Kopriva et al., 2012).

Despite this counterintuitive finding on the positive regulation of *R2R3-MYBs* at S deficiency, the question on the role of such regulation remains open. We suggest that the specificity of the response of different *MYBs* can be achieved by the combinatorial activation of various signaling components acting upstream of *MYBs*. These signaling components include the SLIM1 negatively regulating GSL accumulation, “low IG signal” activating negative feedback loop of IG biosynthesis as well as other known −S-responsive pathways suggested in several studies (Maruyama-Nakashita et al., 2003; Nikiforova et al., 2003) (e.g., the activation of GSL catabolism, production of auxin via IG biosynthesis pathway etc.) and shown in Figure 6A, which build up together a complex regulatory unit for the response of *R2R3-MYB* to S deficiency.

## Materials and methods

### Plant materials and growth conditions in sulfur limitation experiment

Seeds of wild-type *A. thaliana* (Col-0) were grown in a temperature-controlled greenhouse or in a growth chamber in a light/dark cycle of 8 h/16 h at a day/night temperature of 21°C/18°C and 40% humidity.

To analyse the expression of different *MYBs* in response so −S, surface-sterilized seeds of wild type or *cyp79b2 cyp79b3* plants were plated on Hoagland's Media (+S) or corresponding sulfur limiting media (−S) [2 mM Ca(NO_3_)_2_, 0.5 mM KH_2_PO_4_, 0.75 mM MgCl_2_, 10 mM KNO_3_, 1.5 μM CuCl_2_, 2 μM ZnCl_2_, 10 μM MnCl_2_, 50 μM H_3_BO_3_, 0.1 μM MoO_3_, 50 μM KCl, 50 μM Fe-Na-EDTA] or Hoagland's media (Hoagland and Martin, 1950) (+S) [2 mM Ca(NO_3_)_2_, 0.5 mM KH_2_PO_4_, 0.75 mM MgSO_4_, 10 mM KNO_3_, 1.5 μM CuSO_4_, 2 μM ZnSO_4_, 10 μM MnSO_4_, 50 μM H_3_BO_3_, 0.1 μM MoO_3_, 50 μM KCl, 50 μM Fe-Na-EDTA] After 7 and 28 days of growth, the plants were harvested for the analysis of gene expression and GSL content.

### RNA extraction and expression analysis

The isolation of RNA, first strand synthesis and qRT-PCR was performed as described recently (Dean and Annilo, 2005). Relative quantification of expression levels was performed using the comparative ΔΔCt method and the calculated relative expression values were normalized to *Actin2* and to wild-type expression levels (wild type = 1). Primers used for the qRT-PCR analysis are shown in Supplemental Table 1.

### HPLC analysis of desulfo-GS

The isolation and analysis of GSL content was performed by UPLC (Waters, Eschborn) as described recently (Gigolashvili et al., 2012).

### Growth of *Arabidopsis thaliana* cell suspension and overexpression of *SLIM1*

*A. thaliana* dark grown suspension-culture cell line was maintained in 50 mL of *A. thaliana* (AT) medium. The AT medium contained 4.3 g/L MS basal salts (Duchefa), 1 mg/L 2,4-dichlorophenoxyacetic acid (2,4-D), 4 mL vitamin B5 mixture (Sigma-Aldrich) and 30 g/L sucrose (pH 5.8). Cells were gently agitated at 160 rpm in the dark at 22°C.

To generate cells transiently overexpressing *SLIM1*, the full length coding sequence of *SLIM1* was amplified from the cDNA and cloned into the Gateway pDONR207 vector (Life Technologies) using primers containing attB1 and attB2 sequences (SLIM1_attB1: gggacaagtttgtacaaaaaagcaggcttcATGGGCGATCTTGCTATGTCCGTAGC and SLIM1_attB2: gggaccactttgtacaagaaagctgggtcAGCTCCAAACCATGAGAAATCATCAC). The obtained clone was recombined with the *pGWB2* to obtain *Pro35S*-*SLIM1*-*pGWB2*, which was used for transient expression assay.

Transformation of dark-grown cultured Arabidopsis cells was performed using the supervirulent *Agrobacteria* strains LBA4404.pBBR1MCS.virGN54D containing *Pro35S*-*SLIM1-pGWB2* as described by Koprivova et al. (2000).

### Promoter *trans*-activation assay with *SLIM1* and promoter of *MYB51* in cultured *A. thaliana* cells

Promoter of *MYB51* gene was generated as reported in Gigolashvili et al. (2007a). To assess the *trans*-activation potential of *SLIM1* against promoter of *MYB51*, the effector construct with *Pro35S*-*SLIM1-pGWB2* and the promoter reporter *uidA* construct driven by the promoters of *MYB51* gene were used. Thus, the effector construct in the supervirulent *Agrobacterium strain*, the anti-silencing *Agrobacteria* strain 19 K (Voinnet et al., 1999) and *ProMYB51-uidA-pGWB3i* constructs were taken from fresh YEB plates, grown overnight, resuspended in 1 mL AT medium and used for cotransfection (Berger et al., 2007). Three clones of *Agrobacterium* were mixed in 1:1:1 ratio and 75 μL of this suspension was added to 3 mL of cultured *A. thaliana* cells. After 4–5 days of co-culturing (in the dark, 22°C, 160 rpm), cells were treated with 100 μL 5-bromo-4-chloro-3- indolyl-β-D-glucuronid acid (X-Gluc) solution for 1 h to overnight at 37°C.

### Conflict of interest statement

The authors declare that the research was conducted in the absence of any commercial or financial relationships that could be construed as a potential conflict of interest.
