# Supplementary material for: Update on the role of R2R3-MYBs in the regulation of glucosinolates upon sulfur deficiency
Source: Front Plant Sci. 2014 Nov 7;5:626. doi: 10.3389/fpls.2014.00626 (PMC4224069; doi:10.3389/fpls.2014.00626)
Supplement: Supplementary file 1 [file Table1.PDF]

Supplementary Material

Supplementary Table 1. Primer sequences for qPCR analysis

Primer name	AGI-number	Primer sequences 5'-3'
Actin2_RL_Fw	At3g18780	ATGGAAGCTGCTGGAATCCAC
Actin2_RL_Rv		TTGCTCATACGGTCAGCGATG
MYB34_RL_Fw	AT5G60890	CACGACTGTCGATAATTTTGGGTT
MYB34_RL_Rv		CATATTGTCATCTTCGTTCCAGGA
MYB51_RL_Fw	AT1G18570	CTACAAGTGTTTCCGTTGACTCTGAA
MYB51_RL_Rv		ACGAAATTATCGCAGTACATTAGAGGA
MYB122_RL_RV	AT1G74080	AACTTCATTGATCGGCGTCAC
MYB122_RL_Fw		ACCTCTTCGAATCTCCCCATC
CYP79F1_RL_Fw	At1g16410	CCATACCCTTTTCACATCCTACTAGTCT
CYP79F1_RL_Rv		GTAGATTGCCGAGGATGGGC
CYP83B1_RL_Fw	At4g31500	GGCAACAAACCATGTCGTATCAAG
CYP83B1_RL_Rv		CGTTGACACTCTTCTTCTCTAACCG
SLIM1_RL_Fw	AT1G73730	TCTGCAGTGTTTGTACATAACCAGG
SLIM1_RL_Rv		AGGGATTGTAGAAGTTGTACCCTGA
